# Estimation of effective connectivity via data-driven neural modeling

**DOI:** 10.3389/fnins.2014.00383

**Published:** 2014-11-28

**Authors:** Dean R. Freestone, Philippa J. Karoly, Dragan Nešić, Parham Aram, Mark J. Cook, David B. Grayden

**Affiliations:** ^1^Department of Medicine, St. Vincent's Hospital Melbourne, The University of MelbourneFitzroy, VIC, Australia; ^2^NeuroEngineering Laboratory, Department of Electrical and Electronic Engineering, The University of MelbourneParkville, VIC, Australia; ^3^Department of Automatic Control and Systems Engineering, University of SheffieldSheffield, UK; ^4^Centre for Neural Engineering, The University of MelbourneParkville, VIC, Australia

**Keywords:** functional connectivity, neural mass model, model inversion, Kalman filter, epilepsy, seizures, parameter estimation, effective connectivity

## Abstract

This research introduces a new method for functional brain imaging via a process of model inversion. By estimating parameters of a computational model, we are able to track effective connectivity and mean membrane potential dynamics that cannot be directly measured using electrophysiological measurements alone. The ability to track the hidden aspects of neurophysiology will have a profound impact on the way we understand and treat epilepsy. For example, under the assumption the model captures the key features of the cortical circuits of interest, the framework will provide insights into seizure initiation and termination on a patient-specific basis. It will enable investigation into the effect a particular drug has on specific neural populations and connectivity structures using minimally invasive measurements. The method is based on approximating brain networks using an interconnected neural population model. The neural population model is based on a neural mass model that describes the functional activity of the brain, capturing the mesoscopic biophysics and anatomical structure. The model is made subject-specific by estimating the strength of intra-cortical connections within a region and inter-cortical connections between regions using a novel Kalman filtering method. We demonstrate through simulation how the framework can be used to track the mechanisms involved in seizure initiation and termination.

## 1. Introduction

This paper presents a model-based framework for imaging neural dynamics from electrophysiological data. This paper builds on a rich history of research in computational neuroscience that has been increasingly focused on the development of generative models to understand the link between neural activity and neuroimaging data (David et al., 2004; Coombes and Terry, 2012; Moran et al., 2013), with emphasis on two main areas. The first area of focus is forward modeling, or the mapping of relevant neuronal variables to recorded data that facilitates the development of theoretical predictions. The second area of focus is inverse modeling, which is the prediction of states, parameters and neuronal outputs given measured data (David, 2007). The new research presented in this manuscript provides a framework that contributes to solving the inversion problem. A key contribution of this paper is the development of an estimation scheme that is applicable to many alternate neural architectures that can be described by a core set of equations, which encapsulates our knowledge of the biophysics of large-scale neural systems.

Large-scale neural models can combine information from multiple neuroimaging modalities (fMRI, EEG, MEG, etc.), allowing a systems approach for data analysis. The behavior of such models is described by system states, whose dynamics are set by parameters, which are static variables. The systems approach of conducting analyses allows one to study all interactions as a whole. This has advantages over correlation-based science, where correlations do not necessarily reveal causation in large-scale systems. A systems approach provides a unified picture of both local properties and remote interactions, and is considered critical to form an understanding of many of the brain's activities (Freeman, 1975; Deco et al., 2008) including seizure generation (Wendling et al., 2000; Breakspear et al., 2006), which is the focus of this study. In the context of this study, the local properties are described by the connectivity strengths between neural subtypes within the circuitry of a functional processing unit (cortical area or cortical column) and the remote interactions are the functional changes that occur between cortical areas.

The definition of cortical connectivity is multi-faceted and is informed by structural, functional and, more recently, model-based experimentation and analysis (Friston, 1994; David et al., 2004). Despite being multi-faceted, it has been hypothesized that the key characteristics of connectivity within functional processing units in the neocortex can be represented at a high level by canonical neural circuits that are repeated throughout the neocortex (Douglas et al., 1989; Douglas and Martin, 2004; Haeusler et al., 2009). These canonical cortical circuits are able to adapt to the specific functional requirements of the brain through temporal and spatial fluctuations in their interrelationships (da Costa and Martin, 2010). The neural mass model (Jansen and Rit, 1995) that is used for inferring connectivity in this current study can be considered a simplified form of a canonical cortical circuit.

For biological systems, structure is usually a good starting point to study functional interactions (Crick and Koch, 2005). For the brain, this process usually starts with building a map of the anatomic pathways (Sporns, 2013; Van Essen et al., 2013). Often quite separately from the anatomical data, functional relationships are also analyzed through temporal correlations in neuroimaging data, which is recorded from spatially distinct regions of the brain. For example, PET, fMRI, and EEG data have all been used to infer connectivity within and between regions of cortex using a variety of quantitative measures (Biswal et al., 1995; Horwitz et al., 1995; Bokde et al., 2001; Horwitz, 2003). A major challenge lies in consolidating the anatomical data and the functional data to form a unified causative model. This challenge is addressed by the framework presented in this paper.

This paper is concerned with the investigation of effective connectivity through causal modeling. In the context of this paper, effective connectivity is defined as the influence one neural area has on another (Friston, 1994). It is anticipated that the use of causal models, which encapsulate our knowledge of the anatomical connectivity and biophysics of neural populations in conjunction with experimental measurements, will provide a more complete picture of how neural connectivity mediates function. The generation of patient-specific models will also be beneficial in a clinical context, providing greater insight into the cause and progression of neurological disorders, such as epilepsy, and enabling treatment regimes to be investigated through computer simulations.

Analysis of mesoscopic neural dynamics through the use of mean-field models has been validated through several alternative approaches. For example, the so-called neural mass model (Wilson and Cowan, 1972; Da Silva et al., 1974; Freeman, 1987) has been able to describe a large range of neural dynamics such as alpha rhythms (Jansen and Rit, 1995), MEG/EEG oscillations (David and Friston, 2003) and epileptic activity (Wendling et al., 2002). Neural mass models can also be easily extended to define additional population types and larger cortical regions (Babajani-Feremi and Soltanian-Zadeh, 2010; Cui et al., 2011; Goodfellow et al., 2011). The aforementioned results motivate the use of the neural mass model as the basis of a canonical cortical circuit. Furthermore, neural mass models offer a reasonable trade-off between biological realism and parsimony, allowing for practical implementation and subsequent inversion. Inversion is the key to using recorded data to estimate the neural states (membrane dynamics of various neural population subtypes) and parameters (defining connectivity strengths). Estimation of system variables provides new information about underlying population dynamics and physiological properties that cannot be directly measured using other neuroimaging methods (without destroying the tissue). For instance, the connectivity strength between neural population subtypes (i.e., pyramidal, spiny stellate and inhibitory interneurons) have been implicated in seizure generation and have also been found to be patient-specific (Wendling et al., 2000; Breakspear et al., 2006; Blenkinsop et al., 2012).

It has previously been demonstrated that a model-based neurophysiological framework can be used to image parameters associated with seizure onset, evolution and termination in an individual epilepsy patient using ECoG data (Freestone et al., 2013). The framework presented in this manuscript builds on this with improvements to the estimation algorithm and an expansion to include multiple brain regions. Numerous other formulations exist for fitting spatially extended mesoscopic neural models to data. For instance, dynamic causal modeling (DCM) is a technique that is often applied to investigate connectivity of neural areas using generative models (Friston et al., 2003; Kiebel et al., 2006). DCM applies Bayesian inference to determine the most probable configuration of model parameters (i.e., neural coupling coefficients) given a window of recorded data. Therefore, the resulting model is contextualized by the experimentally applied stimuli or conditions under which data was generated (Daunizeau et al., 2011). Another approach has been to apply genetic algorithms to search the parameter space of the model for a structure that is optimal for generating the observed data (Wendling et al., 2005; Nevado-Holgado et al., 2012). In relation to the current work, the aforementioned methods of model optimization can be used to initialize the inversion technique outlined in this paper.

The inversion method outlined in this paper is based on the Kalman filter (Kalman, 1960). The model dynamics are assumed to adhere to a Markov process and estimation quantities (states and parameters) are approximated as random variables with Gaussian distributions. For every electrocorticography (ECoG) measurement, the multivariate state and parameter distribution is propagated through the neural population model; then Bayes rule is used to determine the posterior probability distribution of parameters given measured data. In the case of a linear model, this method is known as the augmented Kalman filter, which provides the optimal (minimizing the variance of the estimation errors) unbiased estimate for states and parameters. Various versions of the Kalman filter equations for nonlinear models have been previously applied for model inversion (Voss et al., 2004; Schiff and Sauer, 2008; Deng et al., 2009; Freestone et al., 2011; Aram et al., 2013; Liu and Gao, 2013). However, these studies were based on either simplified field equations or a single region population model. A key advantage of the Kalman filter-based estimation algorithm outlined in this paper over other expectation maximization or genetic algorithm type schemes is the ability to track states and parameters in real time. Tracking in real time provides a greater level of temporal accuracy in the detection of transitions that underly specific neural activity (such as seizure generation). Furthermore, this paper demonstrates a flexible predictive framework that can be readily adapted to alternative forms of the neural population model (that are based on the same fundamental building blocks) in order to reflect our most current understanding of the architecture of the brain.

The organization of this paper is as follows. The first section outlines the formulation of the computational model of multiple cortical regions and the algorithm for tailoring the model to subject-specific data. Next, example simulations and results are provided that validate the framework for both single and multiple cortical areas. We then provide an example specific to studying epilepsy, where we show how the framework can be used to identify a seizure onset site and the mechanism for seizure initiation and termination. The final section discusses the benefits of this approach in a wider context of understanding seizures and developing much needed new therapies as well as the current limitations of the proposed framework and directions for further work.

## 2. Materials and methods

This section discusses the core biophysics of the mass action of the cortical regions that are incorporated into our mathematical model along with the algorithm for tailoring the model to subject-specific data. Together, the mathematical model and the estimation algorithm form a lens that focuses on the parameters that govern connectivity and function of neural networks.

### 2.1. Neural population model

The neural population model that is used for the framework is based on the neural mass model. This type of neural model describes the dynamics of the mean membrane potential of a population of a specific neuron subtype given firing rate inputs. Populations of this type with varied parameters can be connected together to form local networks to describe the dynamics of specific cortical regions, such as a cortical column. Multiple cortical regions can then be interconnected to form a large-scale network model. Within this section, the building blocks of all neural populations of our large-scale network model are presented that describe the action of the synaptic connections (mean firing rate to mean membrane potential) and the action of the somas (mean membrane to firing rate). The notation used to derive the neural population model in the following section is summarized in Table 1.

**Table 1:** **Notation for the neural population model**.

**Notation**	**Interpretation**
α_*mn*_	Connectivity parameter, population *m* to *n*
*v*_*mn*_	Post-synaptic potential
*z*_*mn*_	Derivative of post-synaptic potential
*v_n_*	Net mean membrane potential for population *n*
*h*_*mn*_(*t*)	Post-synaptic response kernel
ϕ_*m*_	Mean firing rate
*g*(·)	Sigmoidal activation function
*u*	Input from external unmodeled population
τ_*mn*_	Synaptic time constant
ς	Standard deviation of firing thresholds
*v*_0_	Mean firing threshold
*M*	Total number of populations in the model
*N*	Total number of intra-region connections
*J*	Total number of regions in the model
*K*	Total number of inter-region connection
δ	Time step

#### 2.1.1. Single population model

To derive a population model, we begin by defining the mean membrane potential of a neural population, *v_n_*, as the sum of contributing mean post-synaptic potentials, *v*_*mn*_, where the post-synaptic and pre-synaptic neural populations are indexed by *n* and *m*, respectively,

(1) $$v_{n}=\sum_{m=1}^{M}v_{mn}.$$

Each post-synaptic potential arises from the convolution of the input firing rate, ϕ_*m*_(*t*), with the post-synaptic response kernel

(2) $$v_{mn}(t)=\alpha_{mn}\int_{-\infty }^{t}h_{mn}(t-t^{\prime })\phi_{m}(t^{\prime })\text{d}t^{\prime },$$

where α_*mn*_ is a lumped connectivity parameter that incorporates the average synaptic gain, the number of connections and the average maximum firing rate of the presynaptic populations. All lumped connectivity parameters are assumed to be unknown, so must be inferred from data. The post-synaptic response kernels denoted by *h*_*mn*_(*t*) describe the profile of the post-synaptic membrane potential of population *n* that is induced by an infinitesimally short pulse from the inputs (like an action potential). The post-synaptic response kernels are parameterized by the time constant τ_*mn*_ and are given by

(3) $$h_{mn}(t)=\eta (t)\frac{t}{\tau_{mn}}\exp\left(-\frac{t}{\tau_{mn}}\right),$$

where η(*t*) is the Heaviside step function. Typically, α_*mn*_ and τ_*mn*_ are assumed to be constants (particularly for current-based synapses) that define the presynaptic population type. For example, GABAergic inhibitory interneurons typically induce a higher amplitude post-synaptic potential with a longer time constant than glutamatergic excitatory cells. For the model that we are considering, the index *n* (post-synaptic) may represent either pyramidal (*p*), excitatory interneuron (spiny stellate) (*e*) or inhibitory interneuron (*i*) populations.

The inputs to the population, ϕ_*mn*_, may come from external regions, *u*, or from other populations within the model, *g*_*mn*_(*v_m_*), where

(4) $$\phi_{m}=\{\begin{array}{ll} u_{m} & \text{if }m\text{ indexes external inputs}\\ g(v_{m}) & \text{if }m\text{ indexes internal inputs} \end{array}.$$

The various populations within the model are linked via the activation function, *g*(·), that describes a mean firing rate as a function of the pre-synaptic population's mean membrane potential. The activation function exploits a sigmoidal relationship (limited firing rate due to refractory period of the neurons) between the mean membrane potential and firing rate of each of the populations. This sigmoidal nonlinearity may take different forms, but for this study the error function form is used where

(5) $$g(v_{m})\;=\frac{1}{\sqrt{2\pi }\varsigma }\int_{-\infty }^{v_{m}}\exp\left(-\frac{{\left(z-v_{0}\right)}^{2}}{2\varsigma^{2}}\right)\text{d}z$$

(6) $$=\frac{1}{2}\left(\text{erf}\left(\frac{v_{m}-v_{0}}{\sqrt{2}\varsigma }\right)+1\right).\;$$

The quantity ς describes the slope of the sigmoid or, equivalently, the variance of firing thresholds of the presynaptic population (assuming a Gaussian distribution of firing thresholds). The mean firing threshold relative to the mean resting membrane potential is denoted by *v*_0_(*v*_0_ = *v_thresh_* + *v_rest_*). The resting membrane potential is not usually explicitly defined for forward models of this type. However, for inverse models, it is important to understand how the resting membrane potential is included in the equations. The parameters of the sigmoidal activation functions, ς and *v*_0_, are usually assumed to be constants.

The convolution in Equation 2 can conveniently be written as two coupled, first-order ordinary differential equations, which is a second-order state-space model. This gives the system

(7) $$\begin{array}{l} \;\frac{\text{d}v_{mn}}{\text{d}t}=z_{mn}\\ \frac{\text{d}z_{mn}}{\text{d}t}\;=\frac{\alpha_{mn}}{\tau_{mn}}\phi_{mn}-\frac{2}{\tau_{mn}}z_{mn}-\frac{1}{\tau_{mn}^{2}}v_{mn}. \end{array}$$

In summary, this single neural population model maps from a mean pre-synaptic firing rate to a post-synaptic potential. The terms that are usually considered parameters of the model are the synaptic time constants, τ, the connectivity constants, α, the mean firing thresholds, *v*_0_, and firing threshold variances, ς. These parameters can be set to describe connections between specific neural populations, such as pyramidal neurons, spiny stellate cells and fast and slow inhibitory interneurons.

#### 2.1.2. Multiple populations for a cortical region

**Figure 1:**
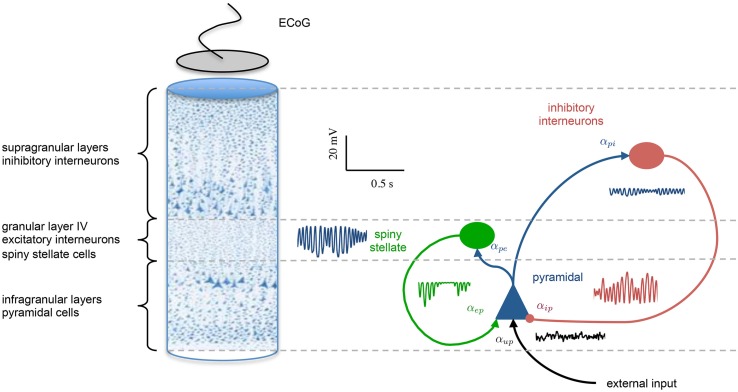
**Population model of a cortical region**. The left hand side shows a cross section of the cortical laminar, highlighting the stratification and different population the various layers. A graphical representation of the population model is presented on the right hand side, showing three interconnected neural populations, which are inhibitory interneurons (supragranular layers), excitatory spiny stellate cells (granular layer), and pyramidal neurons (infraganualar layers). The specific subtype of neural population is defined by the parameters that describe the post-synaptic response kernels. The intra-region connectivity are denoted by α_*mn*_, where the subscript denotes a connection from population *m* to *n*. An example of the post-synaptic potentials that are generated at each connection are also shown.

Multiple populations in the form of Equation 7 can be configured and interconnected to represent the circuitry of a cortical region, such as a cortical column. Each synaptic connection in the model is described by the set of coupled first-order ODEs of Equation 7; however, the parameters are connection-specific. Models exist in the literature describing from two to five different neural types with two to thirteen synaptic connections (4th to 26th order) (Da Silva et al., 1974; Wang and Knösche, 2013). Contributions in this regard have been made by David and Friston (2003); Wendling et al. (2002); Jansen and Rit (1995) and others. An illustration of the model of a cortical region used in this study is shown in Figure 1.

The parameters of the neural populations not only define the population type, but also the behavior the model of the cortical region exhibits. For example, for a certain parameter combination, we obtain a model of a cortical region that will generate alpha-wave type activity; for another set of parameters, we obtain a different model that will exhibit epileptic behavior. The parameters used in this study have been determined previously for similar models (Jansen and Rit, 1995) and are shown in Table 2. The parameters to be estimated are the synaptic gain terms, α_*mn*_.

**Table 2:** **Fixed parameter values for the neural population model that are not estimated**.

**Parameter**	**Value**
ς	3 mV
*v*_0_	6 mV
τ_*up*_, τ_*pe*_, τ_*pi*_, τ_*ep*_	10 ms
τ_*ip*_	20 ms
τ_*d*_	30.3 ms
*u_m_*	220
σ^2^_*u*_	5.74
δ	1 ms

#### 2.1.3. Multiple region model

**Figure 2:**
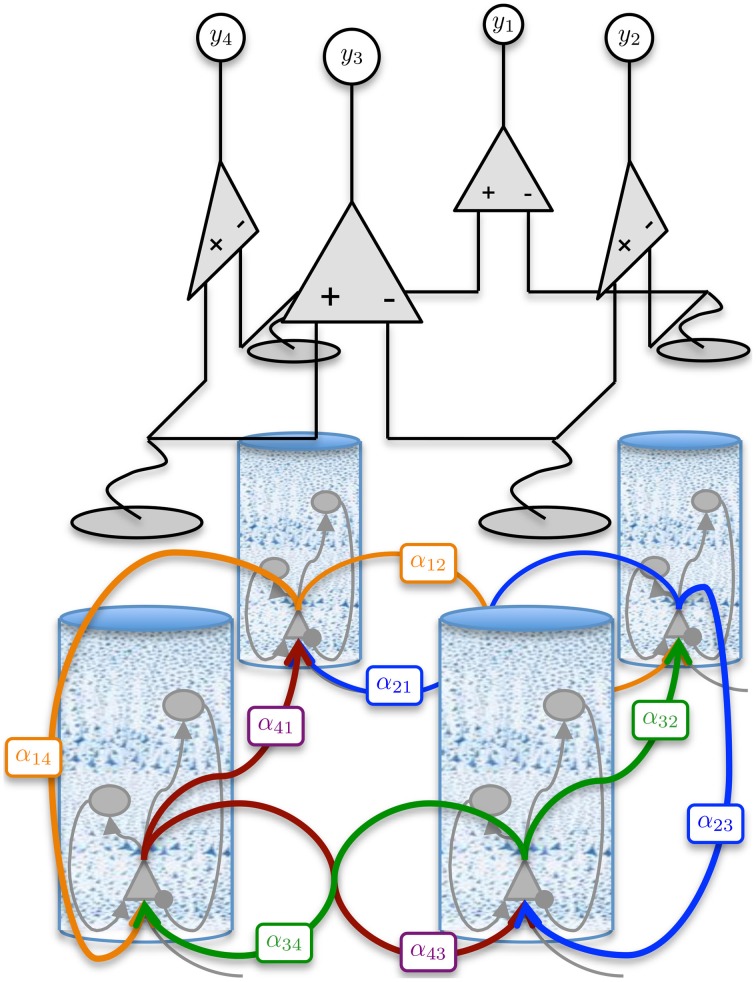
**Graphical representation of the four region population model with differential ECoG measurements**. Each region is interconnected to its immediate neighbor. The inter-region connectivity strength is governed by the parameter α_*jk*_, where *j* and *k* ∈ {1, 2, 3, 4} and *j* ≠ *k*. The differential montage provides a more realistic measurement model then what is typically used for model inversion.

Coupling of cortical region *j* to region *k* is achieved by connecting the output firing rate of the pyramidal population in region *j* to the input of the pyramidal population in region *k* via a delay kernel. The delay kernel is of the same form as the post-synaptic response kernel of Equation 3, but maps a firing rate to a delayed firing rate. The inputs from the delayed firing rates are modeled for every pyramidal population using the same form of second-order model defined in Equation 7. All interconnections between regions were assumed to have the same delay kernel, which was parameterized by a time constant, τ_*d*_ (Wendling et al., 2000) (see Table 2). The delayed firing rates form standard inputs to the pyramidal cells in the adjoining cortical region and induce post-synaptic potentials via a convolution kernel as described by Equation 2. However, the connectivity parameter α_*jk*_ describes the interconnection gain between regions rather than between populations. In this study, we consider four interconnected cortical regions as shown in Figure 2. The values of the interconnection gains for forward simulations were tuned to achieve the desired behavior in the ECoG outputs, while avoiding saturation of neural populations. Different interconnection gains were used to either simulate data consistent with alpha rhythms or to achieve transition to seizure. Further details about the simulations and parameters used are given in Section 2.3.

#### 2.1.4. Augmented discrete time state-space model

For notational convenience, the subscripts for the synaptic gains, denoted α_*mn*_ and α_*jk*_, and the post-synaptic potentials, denoted by *v*_*mn*_ in the previous section, will now be numbered sequentially from 1 to *N* + *K*. *N* is the number of intra-regional connections and *K* is the number of inter-regional connections in the multi-area model.

The state vector is a concatenation of discrete time values of the post-synaptic membrane potentials, the derivatives of the potentials, the delayed firing rates (inter-region) and their derivatives by

$$x≜{\left[\begin{array}{cccccccccc} v_{1} & z_{1} & \ldots & v_{N} & z_{N} & v_{\phi ,1} & z_{\phi ,1} & \ldots & v_{\phi ,K} & z_{\phi ,K} \end{array}\right]}^{⊤},$$

where the large-scale model has *N* intra-region connections and *K* inter-region connections. The subscript ϕ indicates that the post-synaptic potential/derivative is associated with the delayed firing rate from a pyramidal population of a neighboring region.

The parameters to be estimated can also be concatenated into a vector by

$$\theta ≜{\left[\begin{array}{cccccc} \alpha_{l,1} & \ldots & \alpha_{l,N} & \alpha_{d,1} & \;\ldots & \alpha_{d,K} \end{array}\right]}^{⊤},$$

where *l* denotes local connections within regions (including from inputs, *u*), *d* denotes distant connections between regions. For a four-region model, assuming the number of connections within each region is equal, then the number of connections within each region is equal to *N* ÷ 4. In this formulation of the model the parameter vector is written in differential form, with trivial dynamics as

(8) $$\dot{\theta }=0.$$

The differential form of the parameter vector facilitates augmenting the parameters to the state vector for estimation purposes.

The augmented state space vector is created by

(9) $$\xi ≜{\left[\begin{array}{cc} x & \theta \end{array}\right]}^{⊤},$$

which has dimensionality ξ ∈ ℝ^*n*_ξ_^ where *n*_ξ_ = 3(*N* + *K*). The augmented large-scale state space model is given by

(10) $$\dot{\xi }=A\xi +B\xi ◦g\left(C\xi \right)+D\left(u\right)\xi ,$$

where ◦ denotes element-wise multiplication. The matrices **A**, **B**, **C**, and **D**(**u**) are defined in Appendix 5.2. The large-scale model can be written in a compact form that is useful for deriving the estimation algorithm by

(11) $$\dot{\xi }=F\left(\xi ,u\right).$$

It is necessary to discretize the model for estimation purposes. The Euler method was used for discretizing the model and is presented in Appendix 5.1. For the Bayesian inference scheme, it is also necessary to model uncertainty in our model by an additive noise term. With the inclusion of the additive noise term, **w**_*t*_, the discrete time augmented state space model is denoted by

(12) $$\xi_{t+1}=A_{\delta }\xi_{t}+B_{\delta }\xi_{t}◦g\left(C\xi_{t}\right)+D_{\delta }\left(u_{t}\right)\xi_{t}+w_{t}$$

and can be written in compact form by

(13) $$\xi_{t+1}=F_{\delta }\left(\xi_{t},u_{t}\right)+w_{t}.$$

The model uncertainty is defined by a zero mean, temporally white Gaussian with known covariance matrix **Q**. In forward models, **w**_*t*_ is used as a driving term to simulate unknown input to the system from afferent connections or from other cortical regions. However, for model inversion purposes, this additional term also facilitates estimation and tracking of parameters via Kalman filtering or other Bayesian inference schemes. For the Kalman filter, the covariance of **w**_*t*_ quantifies the error in the predictions through the model. If we believed our model is accurate, then we would set all of the elements of **Q** to a small value. On the other hand, a high degree of model-to-brain mismatch can be quantified by setting the elements of **Q** to larger values.

#### 2.1.5. Model of ECoG measurements

It is well accepted that the field potentials that are measured with ECoG are predominately generated by synaptic currents arising from inputs to the pyramidal neurons (Nunez and Srinivasan, 2006). In our model, these currents are linearly proportional to the mean membrane potential of the pyramidal population. Therefore, the ECoG signal is modeled as the mean membrane potential of the pyramidal population, which is the sum of the incoming post-synaptic membrane potentials.

For the multi-region neural population the ECoG measurement is taken to be the difference between neighboring regions. This provides a differential montage that is compatible with experimental data. Typically, the generators of ECoG signals are modeled by the individual mean membrane potentials of the pyramidal populations, effectively ignoring the differential nature of actual ECoG recordings. In this paper, we demonstrate that parameters can be accurately estimating when using the more realistic measurement model.

The measurement model that relates the ECoG measurements to the augmented state vector, ξ_*t*_, is given by

(14) $$y_{t}=H\xi_{t}+v_{t},$$

where **v**_*t*_ ~ 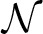
(0, **R**) is a zero mean, spatially and temporally white Gaussian noise process with a standard deviation of 1 mV, that simulates measurement errors. For model inversion purposes, the variance of **v**_*t*_ quantifies the confidence we have in the measurements. The matrix **H** defines a summation of the membrane potentials (corresponding to pyramidal populations) that contribute to each ECoG channel along with the differential montaging scheme. The number of channels used in this case was equal to the number of regions (four), as seen in Figure 2.

### 2.2. A Kalman filter for the population model

The aim of the Kalman filter is to estimate the most likely sequences of states, $\hat{\xi }$^+^_*t*_, and the associated error covariances, $\hat{P}$^+^_*t*_, given (uncertain) knowledge of the biophysics and anatomy of the brain regions of interest combined with the noisy ECoG measurements, **y**_*t*_. The optimal state estimates can be formally stated using the expectations

(15) $${\hat{\xi }}_{t}^{+}\;=𝔼\left[\xi_{t}|y_{1},y_{2},\ldots ,y_{t}\right]\;$$

(16) $${\hat{P}}_{t}^{+}\;=𝔼\left[(\xi_{t}-{\hat{\xi }}_{t}^{+}){\left(\xi_{t}-{\hat{\xi }}_{t}^{+}\right)}^{⊤}\right],$$

which are known as the a posteriori state estimate and state estimate covariance, respectively. The a posteriori state estimate is computed by correcting the a priori state estimate, which is a prediction though our model and defined as

(17) $${\hat{\xi }}_{t}^{-}=𝔼\left[\xi_{t}|y_{1},y_{2},\ldots ,y_{t-1}\right],$$

using a weighted difference between a prediction of the observations and the actual noisy measurements. The a posteriori state estimate is calculated by updating the prediction using measured data by

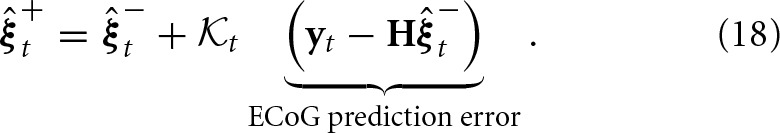



The weighting to correct the a priori augmented state estimate, 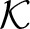
_*t*_, is known as the Kalman gain (Kalman, 1960). The Kalman gain is calculated using the available information regarding the confidence in a prediction of the augmented states through the model and the observation model that includes noise by

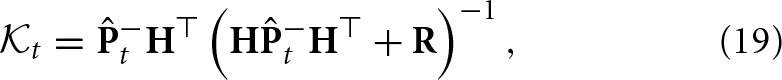



where

(20) $${\hat{P}}_{t}^{-}=𝔼\left[\left(\xi_{t}-{\hat{\xi }}_{t}^{-}\right){\left(\xi_{t}-{\hat{\xi }}_{t}^{-}\right)}^{⊤}\right]$$

is the a priori state estimate error covariance, **R** is the observation noise covariance, and **H** is the observation matrix. For a linear observation function, the a posteriori covariance is then updated by using the Kalman gain to provide the correction

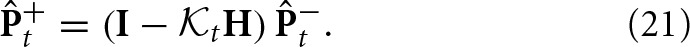



Practically, the actual state is not known so the Kalman filter must be initialized with the best guess for $\hat{\xi }$^+^_0_ and $\hat{P}$^+^_0_, which provides the a posteriori state estimate and state estimate covariance for time *t* = 0. The a priori state estimate for time *t* = 1 can then be computed by propagating the initial guess through the model and taking the expectation,

(22) $${\hat{\xi }}_{t}^{-}\;=𝔼\left[F_{\delta }\left({\hat{\xi }}_{t-1}^{+},u_{t-1}\right)\right]\;$$

(23) $$\;=𝔼\left[A_{\delta }{\hat{\xi }}_{t-1}^{+}+B_{\delta }{\hat{\xi }}_{t-1}^{+}◦g\left(C{\hat{\xi }}_{t-1}^{+}\right)+D_{\delta }\left(u_{t-1}\right){\hat{\xi }}_{t-1}^{+}\right]$$

(24) $$\;=A_{\delta }{\hat{\xi }}_{t-1}^{+}+𝔼\left[B_{\delta }{\hat{\xi }}_{t-1}^{+}◦g\left(C{\hat{\xi }}_{t-1}^{+}\right)\right]+D_{\delta }\left(u_{t-1}\right){\hat{\xi }}_{t-1}^{+}$$

Generally, for nonlinear systems, the solution to this expectation is not known. Therefore, approximations are often used, such as the extended and unscented Kalman filters, respectively.

We approximate the expectation by

(25) $$𝔼\left[B_{\delta }{\hat{\xi }}_{t-1}^{+}◦g\left(C{\hat{\xi }}_{t-1}^{+}\right)\right]\approx B_{\delta }{\hat{\xi }}_{t-1}^{+}◦𝔼\left[g\left(C{\hat{\xi }}_{t-1}^{+}\right)\right],$$

where the accuracy of the approximation depends on the width of the distributions for the parameters, **Bξ**^+^_*t* − 1_. Since we are assuming the parameters are unknown with the possibility of slow changes, a small amount of uncertainty is added. For known parameters, Equation 25 would be exact. Therefore, the accuracy of the approximation improves as parameter estimates converge toward their actual values.

In an effort to improve state and parameter estimation accuracy, a new innovation in this study is an analytic solution to the expectation of the mean membrane potential, which is modeled as a Gaussian, transformed by the sigmoid. To show the solution, we first point out that

(26) $$\gamma_{j}{\hat{\xi }}_{t-1}^{+}={\hat{v}}_{t,j}$$

corresponds to the total pre-synaptic mean membrane potential of the *j*th neural population, where γ_*j*_ is a row vector from the adjacency matrix, **C**, which is described in detail in Appendix 5.2. Also, the variance of the pre-synaptic mean membrane potential is

(27) $$\gamma_{j}{\hat{P}}_{t-1}^{+}\gamma_{j}^{⊤}={\hat{\sigma }}_{t,j}^{2}.$$

The analytic solution for the expectation of a Gaussian distributed random variable (total membrane potential of the respective population) transformed by the sigmoid error function, *g*(·), is given by

(28) $$𝔼\left[g\left(\gamma_{j}{\hat{\xi }}_{t-1}^{+}\right)\right]=\frac{1}{2}\left(\text{erf}\left(\frac{\gamma_{j}{\hat{\xi }}_{t-1}^{+}-v_{0}}{\sqrt{2\left(\varsigma^{2}+\gamma_{j}{\hat{P}}_{t-1}^{+}\gamma_{j}^{⊤}\right)}}\right)+1\right)\text{​​}.$$

The derivation of this new result is shown in Appendix 5.3.

The a-priori covariance is approximated using the unscented transform, which approximates the statistics of a multivariate Gaussian that undergoes a nonlinear transformation (Julier and Uhlmann, 1997). The approximation is given by

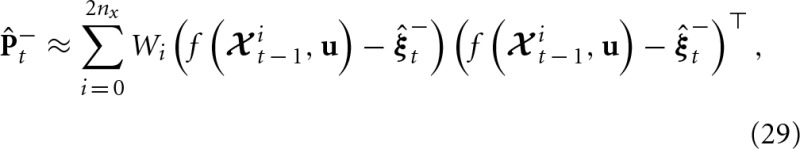



where 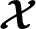
^*i*^_*t* − 1_ is a matrix of sigma vectors, which are carefully chosen samples from the distribution of $\hat{x}$^+^_*t* − 1_, and *W_i_* are vectors of weights associated with the transform. For completeness, the method of computing the sigma vectors and the weights is provided in Appendix 5.4.

It is likely that the parameters and states described by a cortical circuit will be subject to identifiable physiological constraints that should be included in an inversion problem in order to exploit all available information. There are various ways to constrain the parameter space by truncating the distribution of the prior (Simon, 2006). In this study, a computationally simple method known as “clipping” (Kandepu et al., 2008) was used to constrain the synaptic gains. Upper and lower bounds on synaptic gain estimates were enforced during the calculation of the posterior distribution by imposing limits on the analytic calculation of the mean and on the sample space of the unscented transform (used to approximate the covariance). The bounds were set larger than proposed ranges for the intra-regional parameters of a multi-area neural mass model, determined by Babajani-Feremi and Soltanian-Zadeh (2010). The bounds for the constraints are shown in Table 3.

**Table 3:** **Parameter constraints used in the clipping method of the estimation algorithm**.

**Parameter**	**Lower bound**	**Upper bound**
α_*up*_	0	300
α_*ep*_, α_*pi*_, α_*pe*_	0	20,000
α_*ip*_	−40,000	0
α_*jk*_, α_*kj*_	0	5000

### 2.3. Simulations for validation

In order to test the performance capabilities of the model-based framework, it is necessary to use data where the actual parameter values are known. While it is impossible to accurately measure parameter values in an experiment, it is possible to know the actual values when using data that is generated in a forward simulation. Therefore, artificial data was used to test the estimation performance. This type of test does not guarantee that the method will work with clinical recordings, but provides a proof of principal based on the assumption that our neural population model provides a reasonable representation of cortical dynamics. Considering the wide range of phenomena that the population model has been able to describe and the wide acceptance in the literature, this assumption is a reasonable starting point.

In order to test the robustness of the estimation algorithm, a Monte Carlo simulation was performed by testing the estimation algorithm with 50 realizations of synthetic data, each with a different unknown input. For each of the realizations, the parameters were set such that the model generated activity with a dominate spectral peak at around 10 Hz (alpha activity). The parameter values are shown in Table 4. The accuracy of parameters estimates (connectivity gains) are measured in terms of percentage bias and were taken as the absolute difference between the estimated and true values at the end of each simulation. Simulations were run for 60 s for the single-region model and 100 s for the four-region model, as the parameter estimates were observed to converge well within this time. For state tracking, only the results of the post-synaptic potentials are shown, although the derivatives of the post-synaptic potentials were also tracked. State accuracy was measured by the root mean squared (RMS) error over 1 s of data, since the states (and their estimates) are dynamic. The RMS error was measured from the final second of the simulation, when parameter estimates were assumed to be constant. Results are also presented for a single realization for both the single and four region models (normal and epileptiform) in order to illustrate the convergence properties over time of the parameter estimates. The parameters used to simulate the epileptic-type behavior seen in the simulated seizure transition are given in Table 5. The bounds that were used to constrain the parameter estimates are shown in Table 3.

**Table 4:** **Connectivity parameters to simulate an alpha rhythm in the multi-region population model**.

**Parameter**	**Value**	**Parameter**	**Value**
α_*up*_	3.2	α_21_, α_41_	76
α_*ep*_	1755 α_12_, α_32_	63
α_*pi*_	548.4 α_23_, α_43_	44
α_*ip*_	−3712.5	α_14_, α_34_	70
α_*pe*_	2197		

**Table 5:** **Connectivity parameters used to simulate epileptic behavior in the multi-region population model**.

**Region 1**		**Regions 2, 3, 4**		**Interconnectivity**	
α_*up*_	8.1	α_*up*_	3.2 α_21_, α_41_	1.6
α_*ep*_	4387 α_*ep*_	1755	α_12_, α_32_	162.5
α_*pi*_	1370.9	α_*pi*_	548.4 α_23_, α_43_	162.5
α_*ip*_	−3712.5	α_*ip*_	−3712.5	α_14_, α_34_	162.5
α_*pe*_	5483.7	α_*pe*_	2197		

## 3. Results

### 3.1. Comparison of analytic mean and unscented transform

The performance of the modified Kalman filter and the unscented Kalman filter were compared in order to quantify the increase in estimation performance from using the analytic mean. Both methods approximated the covariance of the joint distribution using the unscented transform. Since the mean and covariance cannot be considered separately when the distribution is propagated through the neural population model, the Kalman filter that uses the analytic mean is really an approximation of a Gaussian distribution. However, the difference between the standard UKF and this novel application of the Kalman filter, which is tailored to the neural population model, is that the new approach based on the analytic mean has the potential to improve state and parameter estimation for this particular application.

**Figure 3:**
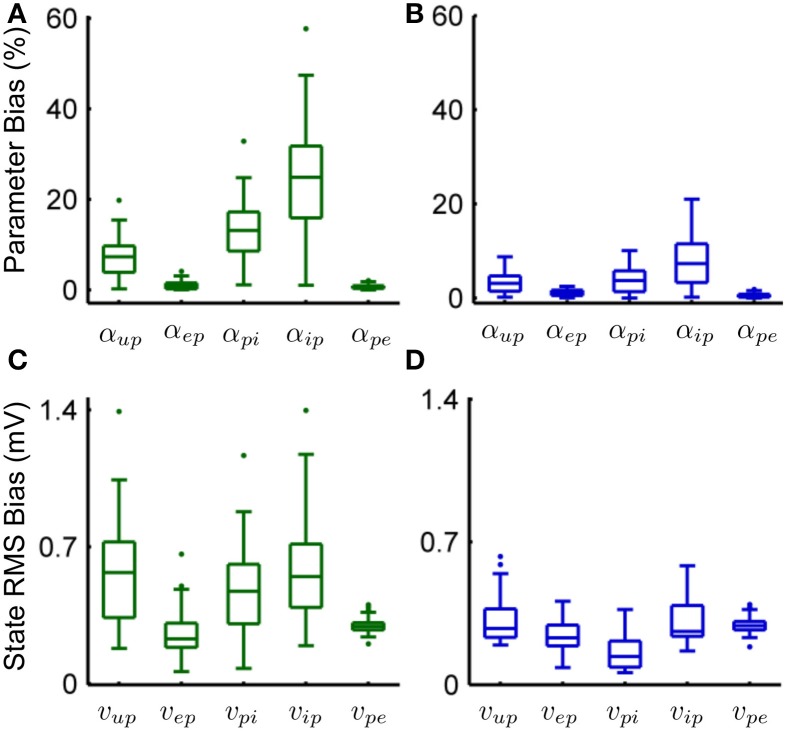
**Comparison of the estimation results from the modified Kalman filter with the unscented Kalman filter from the Monte-Carlo simulation (50 realizations). (A)** The bias for parameter estimation as a percentage of the true value for the connectivity gain using the UKF. **(B)** The bias for parameter estimation as a percentage of the true value for the connectivity gain using the analytic mean. **(C)** RMS error for state tracking of the post synaptic potentials using the UKF. **(D)** RMS error for state tracking of the post synaptic potentials using the analytic mean. The center line of the box plots shows the median error and the box covers are the 25th to 75th percentiles. The whiskers cover the entire range of errors that are not considered outliers, which are shown by the dots. The outliers are determined to be outside *q*_1_ − 1.5(*q*_3_ − *q*_1_) to *q*_3_ + 1.5(*q*_3_ − *q*_1_) where *q*_1_ and *q*_3_ denote the 25th and 75th percentiles.

Tables 6, 7 show the mean estimation bias for intra-connectivity gains and post-synaptic potentials (PSPs) of a single cortical region. Table 6 demonstrates that the analytic mean approach is approximately twice as accurate as the UKF for state tracking of *v*_*up*_, *v*_*pi*_ and *v_ip_* and has equal accuracy with the UKF for *v_ep_* and *v_pe_*. This is consistent with the parameter estimates in Table 7, which shows that the analytic mean method gave two to three times improved accuracy over the UKF for α_*up*_, α_*pi*_ and α_*ip*_ (and has the same accuracy for α_*ep*_ and α_*pe*_). Figure 3 shows the results for the entire Monte Carlo simulation and again demonstrates that the Kalman filter using an analytic mean outperforms the UKF for the single region model. Figures 3A,B show that the intra-connectivity gain estimation is within 60% for all parameters for the UKF and less than 25% for the analytic mean method. Figures 3C,D show that the bias for tracking of PSPs is consistently less than 1.4 mV for the UKF and less than 0.7 mV for the analytic mean approach. On the whole, these results demonstrate the value of the novel application of the modified Kalman filter for the neural population model.

**Table 6:** **Mean bias (over 50 simulations) of the post-synaptic potential estimates for a single region model of alpha rhythms, with comparison between the UKF and the new modified Kalman filter**.

**Post-synaptic potential**	**RMS Bias (mV)**	
	**Unscented transform**	**Analytic mean**
*v*_*up*_	0.57	0.32
*v*_*ep*_	0.26	0.24
*v*_*pi*_	0.47	0.16
*v*_*ip*_	0.58	0.31
*v*_*pe*_	0.30	0.29

**Table 7:** **Mean bias (over 50 simulations) of the connectivity gain estimates for a single region model of alpha-type rhythms, with comparison between the UKF and the new modified Kalman filter**.

**Connectivity gain**	**Bias (%)**	
	**Unscented transform**	**Analytic mean**
α_*up*_	7.33	3.45
α_*ep*_	1.07	1.05
α_*pi*_	13.29	4.01
α_*ip*_	24.01	7.69
α_*pe*_	0.73	0.58

### 3.2. Single region model

**Figure 4:**
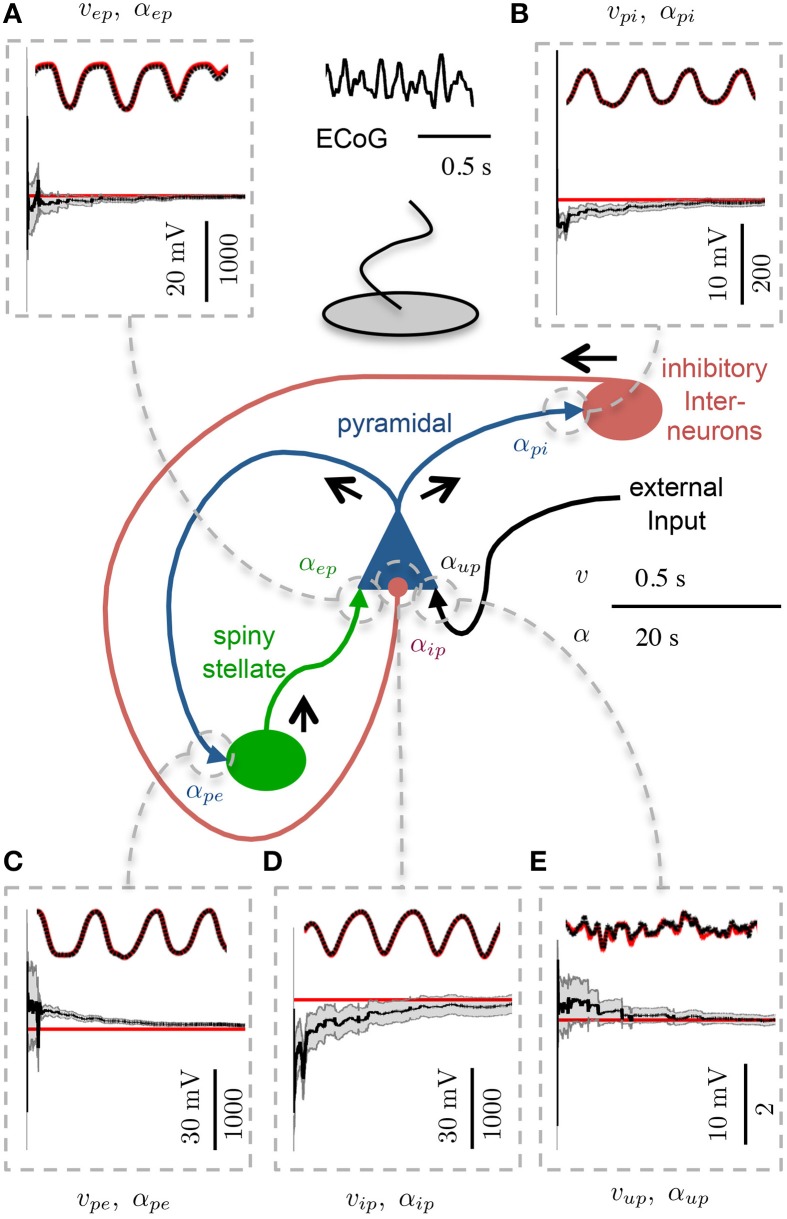
**Estimation results showing convergence of parameters in the single region model**. 30 s of ECoG data simulating an alpha rhythm from a single region model was used. Each panel shows the PSP (upper) and connectivity gain (lower) estimates. The actual states are shown in red and the estimated values are shown in black. The gray shaded regions show the estimated standard deviation estimates of the connectivity gains. The scale in the lower left of each subpanel is distinct for the PSP (LHS) and connectivity gain (RHS) **(A)** PSP and connectivity gain for spiny stellate to pyramidal connection. **(B)** PSP and connectivity gain for pyramidal to inhibitory interneuron connection. **(C)** PSP and connectivity gain for pyramidal to spiny stellate connection. **(D)** PSP and connectivity gain for inhibitory interneuron to pyramidal connection. **(E)** PSP and connectivity gain for external input to pyramidal connection.

Figure 4 shows an example of state tracking and parameter estimation for a single cortical region. The plots show that the algorithm was able to reliably track all postsynaptic potentials and estimate all connectivity gains in the region. This remarkable result was achieved using only the noisy ECoG signal and knowledge of the structure of the cortical circuit. Figure 4 also shows that the standard deviation of the estimated parameters also converged, which demonstrates the filter was performing as expected. The standard deviation of the estimate for α_*ip*_ remained larger than the estimates for the other connectivity gains, as it had the largest bounds representing greater uncertainty.

Figures 3B,D show the results for parameter estimation and state tracking using the Kalman filter with the analytic mean for a Monte Carlo simulation with 50 realizations. Both figures demonstrate good accordance for estimation results to the actual states and parameters, with the possible exception of the inhibitory-to-pyramidal connectivity gain estimate (α_*ip*_) when using the standard unscented Kalman filter.

From Figure 3D and Table 6 it can be seen that the bias of the state (PSP) tracking was consistently less than 0.7 mV and the mean RMS bias was less than 0.4 mV for all the potentials when using the modified filter. The amplitude of the PSPs was on the order of 10–30 mV, thus an average bias of less than 0.4 mV represents satisfactory performance. The tracking of post-synaptic potential induced from the input, *v*_*up*_, was the worst performer. This is to be expected since it is linked to the connection from the stochastic input, *u*(*t*), and the pyramidal population. Figure 3B and Table 7 show that the mean estimation bias for all of the connectivity coefficients (slow states) was less than 22% with a mean of less than than 8%. It is anticipated that this level of accuracy in state estimation will provide a strong basis for a classification algorithm that distinguishes between healthy and abnormal oscillations (such as observed during seizures).

### 3.3. Four region model

**Figure 5:**
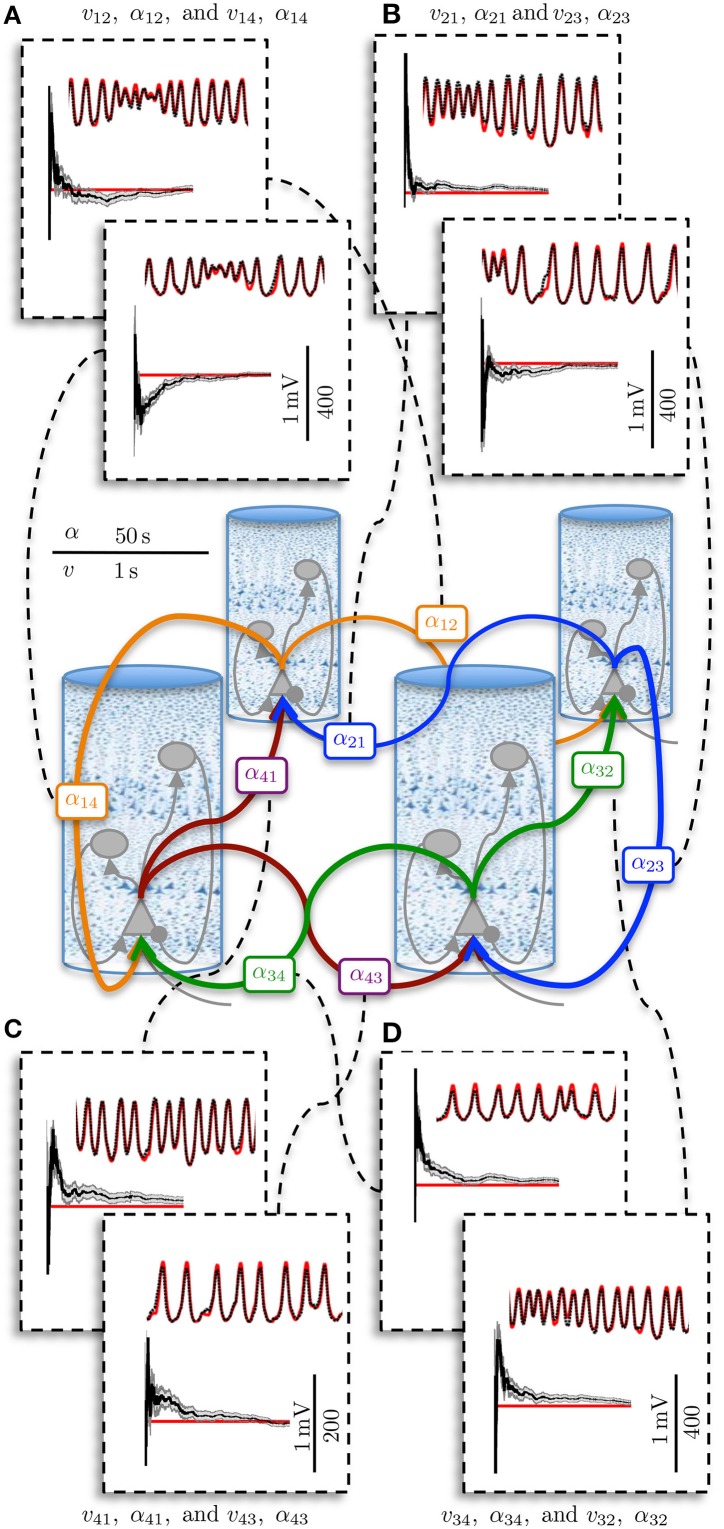
**Post-synaptic potential and connectivity gain estimation results for the four region model showing parameter convergence**. ECoG data was obtained over a 50 s simulation using the four region model to generate alpha-type rhythms. The filter output for PSP tracking is over a short time segment and the connectivity gain estimation is for the entire simulation. The actual states are shown in red and the filter output is shown in black. The gray bar around the plot of the connectivity gain estimates shows the standard deviation of the estimate. **(A)** PSP and interconnectivity gains from region one to two (upper) and four (lower). **(B)** PSP and interconnectivity gains from region two to one (upper) and three (lower). **(C)** PSP and interconnectivity gains from region four to one (upper) and four to three (lower). **(D)** PSP and interconnectivity gains from region three to four (upper) and three to two (lower).

Figure 5 shows an example estimation result for the four region model. The four region model has four times as many measurements that are inputs to the filter, as there are additional ECoG voltage signals (one per region). However, the dimensionality of the system is more than four times larger than the single column, as each new column introduces an equal number of intra-regional connections as well as two inter-regional connections with its neighbors. In Figure 5, only the inter-regional connections are shown, although all of the PSPs and connectivity gains were estimated. The results that are presented in Figure 5 demonstrate that the estimation method was capable of scaling up from a single region model to a larger model of coupled regions, while maintaining the ability to simultaneously estimate all the connectivity gains and track the PSPs associated with every synapse. The ability to scale up to a larger area is crucial in order to apply estimation to patient-specific models of epilepsy.

**Figure 6:**
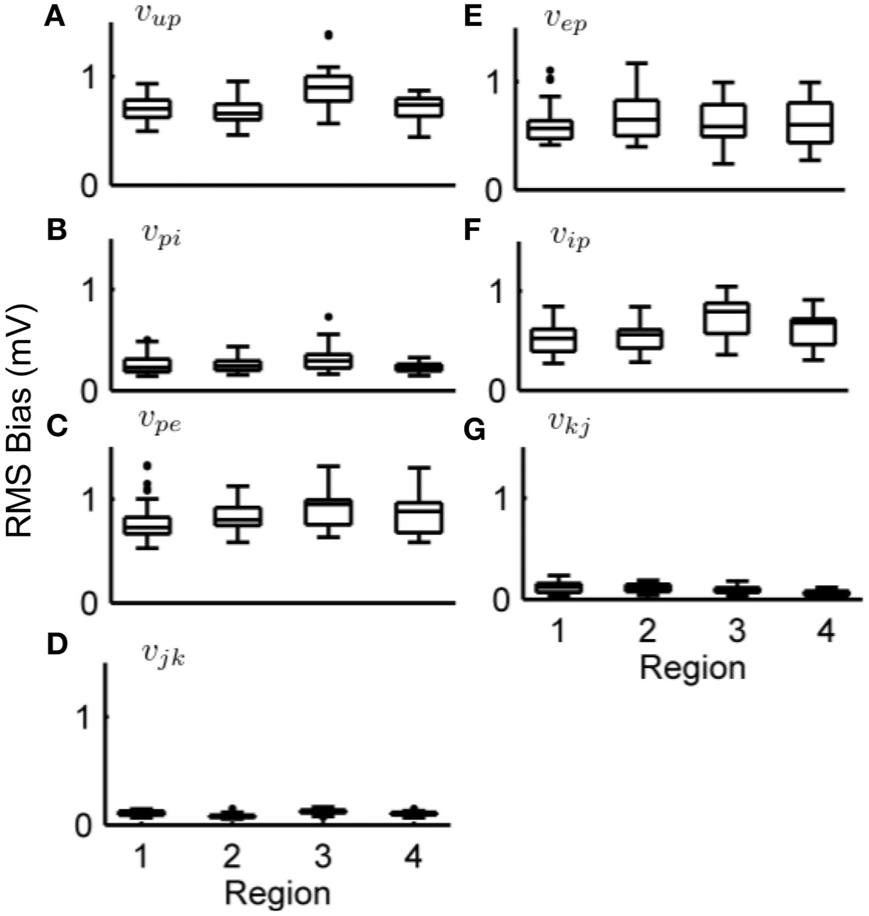
**Post-synaptic potential estimation results in the four region model from a Monte-Carlo simulation**. Each subplot shows the RMS bias for state tracking of a PSP associated with a specific synapse over 50 simulations. **(A)** RMS bias for *v*_*up*_. **(B)** RMS bias for *v*_*pi*_. **(C)** RMS bias for *v_pe_*. **(D)** RMS bias for *v_jk_*. **(E)** RMS bias for *v_ep_*. **(F)** RMS bias for *v_ip_*. **(G)** RMS bias for *v_kj_*. ECoG data was obtained using the four-region model generating alpha-type rhythms, with different stochastic input for every simulation. For every subplot, the centerline of the boxplots are the median and the edges are the 25th and 75th percentiles. Outliers are determined to be outside *q*_1_ − 1.5(*q*_3_ − *q*_1_) to *q*_3_ + 1.5(*q*_3_ − *q*_1_) where *q*_1_ and *q*_3_ denote the 25th and 75th percentiles.

**Figure 7:**
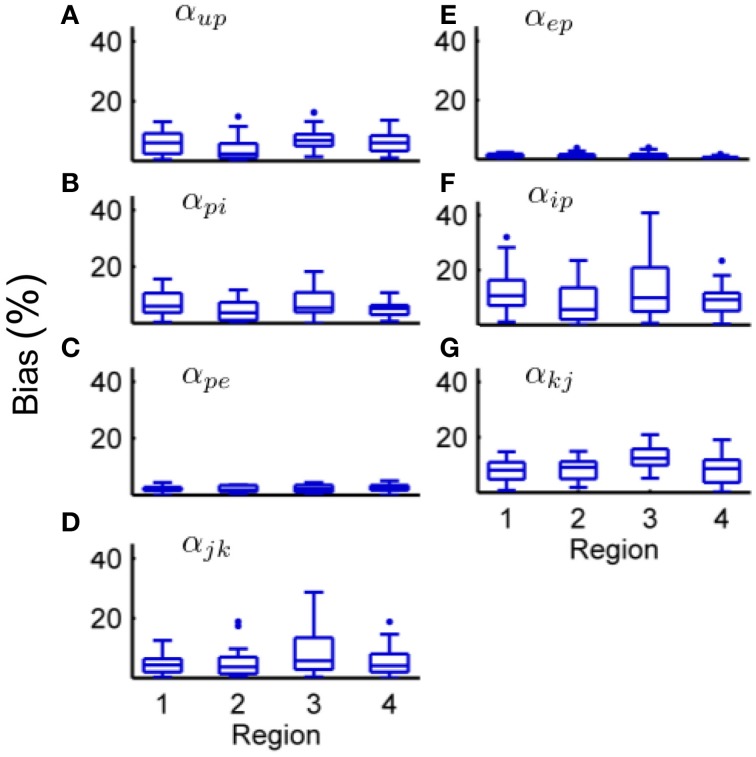
**Connectivity estimation results in the four region model from a Monte-Carlo simulation**. Each subplot shows the estimation bias as a percentage of the true value for the connectivity gain for every synapse over 50 simulations. **(A)** Bias for α_*up*_. **(B)** Bias for α_*pi*_. **(C)** Bias for α_*pe*_. **(D)** Bias for α_*jk*_. **(E)** Bias for α_*ep*_. **(F)** Bias for α_*ip*_. **(G)** Bias for α_*kj*_. ECoG data was obtained using the four-region model generating alpha-type rhythms, with different stochastic input for every simulation. For every subplot, the centerline of the boxplots are the median and the edges are the 25th and 75th percentile. Outliers are determined to be outside *q*_1_ − 1.5(*q*_3_ − *q*_1_) to *q*_3_ + 1.5(*q*_3_ − *q*_1_) where *q*_1_ and *q*_3_ denote the 25th and 75th percentiles.

Figures 6, 7 show the estimation bias over 50 simulations for the connectivity gains and PSP tracking, respectively. Each simulation was run for 100 s (as in Figure 5) with a different randomly generated sequence for *u*(*t*) as external input. Tables 8, 9 summarize the mean (over the 50 simulations) values of the estimation biases for both fast and slow states. Figure 6 and Table 8 show that the RMS bias for PSP tracking was consistently less than 1.5 mV and the mean RMS bias was less than 1 mV for all connections. The amplitude of the PSP signals was on the order of 10–30 mV and the variance of noise added to the ECoG voltages was 1 mV. Therefore, the bias for PSP tracking represents a high level of accuracy. As was seen for the single region model, the tracking performance was less accurate for *v*_*up*_ due to the stochastic input that generates this PSP.

**Table 8:** **Mean RMS estimation bias (over 50 realizations in mV) for post-synaptic potential tracking in the multi-region model**.

	**R1**	**R2**	**R3**	**R4**
*v_up_*	0.72	0.71	0.91	0.71
*v*_*ep*_	0.51	0.61	0.74	0.57
*v*_*pi*_	0.78	0.88	0.95	0.84
*v*_*ip*_	0.63	0.74	0.74	0.62
*v*_*pe*_	0.26	0.26	0.32	0.24
*v*_*jk*_	0.14	0.13	0.11	0.07
*v*_*kj*_	0.19	0.15	0.12	0.2

**Table 9:** **Mean bias (over 50 realizations in %) for connectivity parameter estimates in the multi-region model**.

	**R1**	**R2**	**R3**	**R4**
α_*up*_	6.11	3.6	7.32	6.15
α_*ep*_	1.05	1.24	1.35	0.63
α_*pi*_	6.87	4.01	6.68	4.91
α_*ip*_	12.21	7.62	13.02	9.14
α_*pe*_	1.94	2.16	2.06	2.58
α_*jk*_	7.76	8.28	12.92	8.35
α_*kj*_	4.48	4.81	8.01	4.94

Figure 7 and Table 9 show that the estimation bias for the connectivity gains was less than 40% and the mean bias was less than 10%, except for α_*ip*_ and α_*jk*_ which were less than 15%. The parameter estimation accuracy for the coupled model compared with the single region model was comparable in terms of the mean value for all connectivity gains. Over the entire Monte Carlo simulation, the estimation performance for α_*ep*_, α_*pi*_ and α_*pe*_ were similar to the single region model. The decrease in performance is most evident for α_*ip*_ (from within 20% to within 40%). This is consistent with the results from the single region model where α_*ip*_ was the least accurate of the estimated gains. The estimation performance for α_*jk*_ and α_*kj*_ cannot be compared to the single region model. However, the estimation accuracy of the interconnectivity gains was worse than the intra-region gains (apart from α_*ip*_). It is difficult to pinpoint sources of error for this parameter, as all of the estimated states are highly interactive with each other. A potential source of the decreased accuracy for α_*jk*_ and α_*kj*_ (as well as α_*up*_) is that their values are an order of magnitude smaller than the other estimated connectivity gains, which can lead to numerical problems for the Kalman filter equations. On the whole, the consequences of scaling up the model from a single region to four coupled regions has not resulted in major loss of estimation accuracy.

### 3.4. Simulation of an epileptic seizure

**Figure 8:**
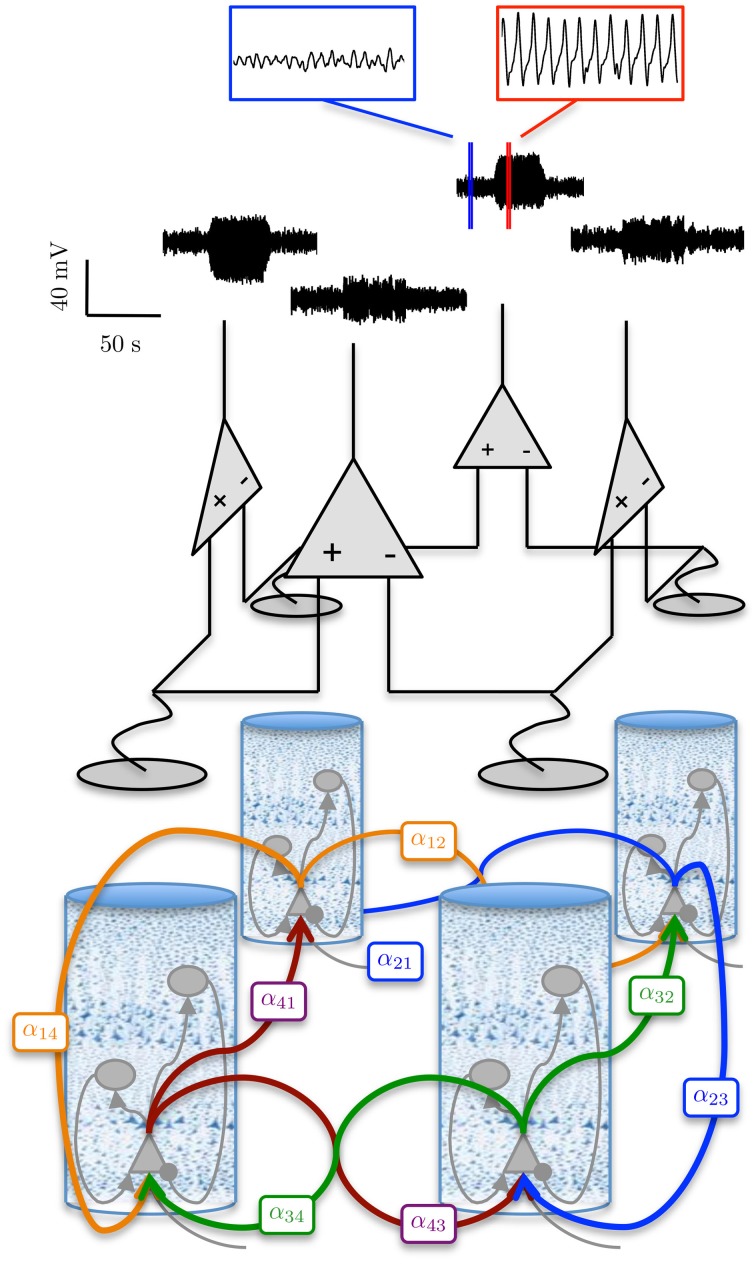
**Simulation of an epileptiform transition**. ECoG signals were obtained using a 100 s forward simulation and adjusting the connectivity gains from alpha to seizure rhythms and vice versa (see Tables 4, 5). The simulation output shows the epileptiform activity rapidly spreading from Region 1 (where the pathology was simulated), to the rest of the network. The figure also shows a graphical representation of the model of the differential measurement function. The blue and red sub-panels show example alpha and seizure-type rhythms, respectively.

Figure 8 shows a simulated ECoG time series with transitions from a background rhythm to seizure-like oscillations and back. The transitions were achieved in the forward simulation by ramping the amplitude of the excitatory gains of one cortical region (region 1 in Figure 8) and then decreasing them back to their usual values. The values used to generate the seizure-type behavior are shown in Table 5. In order to ensure that the seizure-like oscillations would spread from one region to the neighboring regions, the interconnectivity between the first area (where the seizure was initiated) to its neighbors was increased from the previous example over the entire time course of the simulation, while the interconnectivity gains from all other regions back to the first region were decreased (as shown in Table 5).

**Figure 9:**
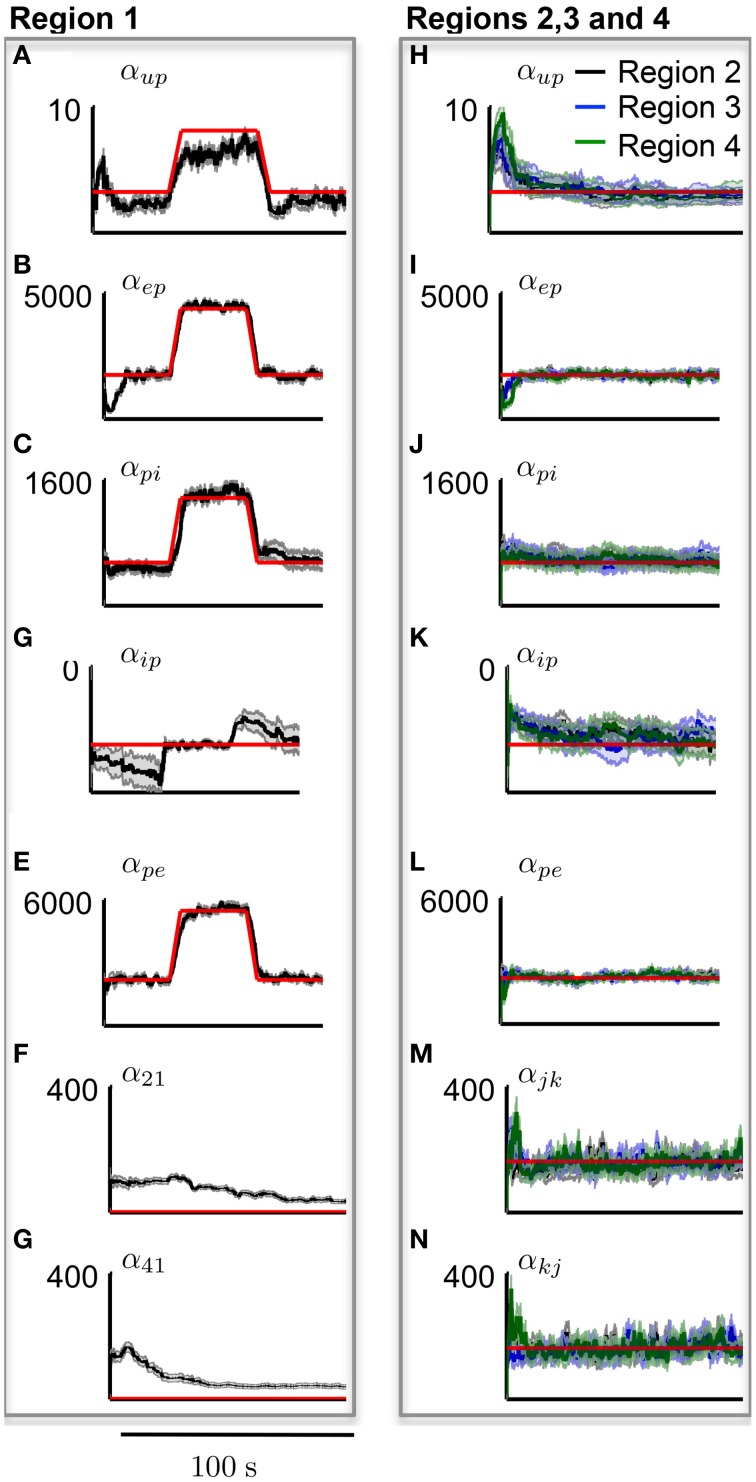
**Results from tracking pathological changes in the connectivity gains that lead to epileptiform activity**. In each subplot, the red line shows the actual values. **(A–G)** Show the estimation results from Region 1, where the internal excitatory connectivity gains were transiently increased to induce the epileptiform discharge. The mean is shown by the black line and the gray shaded area shows the standard deviation of the estimate. **(H–N)** Show the estimates from the non-pathological regions (no change in parameters from baseline), where the solid lines show the mean and shaded regions show the standard deviation of the parameters.

Figure 9 shows the estimation results of the connectivity gains for each cortical area during the simulated seizure. In order to track parameter changes (compared with the previous estimation when parameters were assumed to be static), additional uncertainty was added to the estimate error covariance in the Kalman filter (see Appendix 5.4.). The additional uncertainty was required to inflate the estimation error covariance to capture unmodeled transitions in parameter values. It is clear that the method has successfully identified the transitions in the cortical region that led to the seizure generation, as the filter tracked the increase in these gains for region 1, while accurately estimating the corresponding connectivity gains for the other cortical regions that remained constant.

It can be seen from Figure 9A that the estimation accuracy for α_*up*_ was lower than the other connectivity gains due to the stochastic input. The estimated interconnectivity gains that were associated with inputs to region 1 (the epileptic region), α_21_ and α_41_, also do not quite converge (Figures 9F,G) the actual values. This could be due to the much smaller magnitude of these gains compared with the corresponding interconnectivity gains in the other regions. From Figure 9D, it can also be noted that the estimation accuracy of inhibitory to pyramidal connectivity, α_*ip*_, did not converge to the actual value in first part of the simulation (alpha rhythm), which was also consistent with previous results. However, the estimates of α_*ip*_ converged to actual values during the seizure and had a lower estimation standard deviation, which can be attributed to the higher signal-to-noise ratio during larger amplitude oscillations. If this method of estimation can be translated for use on real data, it has the potential to provide valuable insight into the cause and spread of seizures and enable more informed treatment measures for epilepsy patients.

## 4. Discussion

This paper presented a framework for model inversion that facilitates estimation and imaging of the physiological properties of the brain using electrocorticography (ECoG) data, under the assumption that the model captures the key features of the cortical circuits of interest. Tracking of the mean membrane potentials of the various neural populations and connectivity parameters (within and between cortical regions) may provide a clear picture of the causal relationships between cortical dynamics and seizures. The link between physiological parameters and data will undoubtedly improve detection and treatment outcomes across a range of pathologies.

We have demonstrated that is possible to reliably track the post-synaptic potentials and estimate the connectivity parameters of a large-scale neural population model. This demonstration highlights the power of combining the prior information we have about neural dynamics and cortical structure (that is encoded in the computational model) to estimate the parameters of interest. For the single region case, the average prediction bias for connectivity parameters is less than 8% and the average RMS error in the mean post-synaptic potential estimates within the local circuit was less than 0.4 mV (the peak to peak potential of a typical post-synaptic potential was approximately 20 mV). We demonstrated that the framework can be scaled up to a larger-scale model (of four cortical regions) with more realistic measurements without a major decrease in estimation accuracy. The average estimation error remained less than 10% except for three parameters (errors in α_*ip*_, α_*jk*_, and α_*kj*_ were less than 15%). The tracking of post-synaptic potentials in the four-region model had mean RMS error of less than 1 mV. Importantly, we demonstrated the ability to track slow changes in the connectivity parameters, that led to transitions to and from seizures. Traditionally, functional neuroimaging methods have been very successful, but limited to determining where and when seizures occur. This new method can be used with ECoG data to also determine the mechanisms. This knowledge will provide opportunities to develop new therapies.

Traditionally, amplitude, frequency and phase correlations in neuroimaging data have been used as features to study connectivity. While these techniques imply a causal relationship, they can be misleading. For instance, correlations that arise between multiple microelectrode neural recordings could be the result of neurons independently responding to a common stimulus or could be caused by synaptic coupling between neural populations (Friston, 1994). Other possibilities that need to be taken into account are neural populations receiving a common modulatory input from another unobserved region of the brain, or indirect coupling between neural populations where connectivity is affected via multiple regions (Friston, 1994). Questions about the sources of correlation in neural recordings are difficult to disambiguate without resorting to more invasive methods of measurement. On the other hand, computational models can directly infer cortical connectivity patterns and neural dynamics from data, providing the probable cause of empirical observations. The degree to which such causal relationships correspond to the true state of the cortex is limited by the model uncertainty, just as correlations identified using other types of neuroimaging are limited by spatial and/or temporal resolution constraints. However, model uncertainty can be quantified, which is a highly useful property for many classification applications.

Under a Gaussian assumption, the Kalman filter provides estimates of the probability distributions of the states and parameters of the population model, which is updated as new measurements become available. If the Gaussian assumption holds, the Kalman filter provides the minimum variance estimate of the states and parameters (Simon, 2006). However, the nonlinearities in the model lead to non-Gaussian states. Nevertheless, the Gaussian approximation leads to good estimation results, as demonstrated by the Monte Carlo simulations. However, these results do not guarantee that the state and parameter estimates will not eventually diverge from the actual values, given a measurement times series of a longer duration. This is due to the approximations of the unscented transform. Possible improvements in the estimation results could come from using sequential Monte Carlo (SMC) filtering methods, when the Gaussian assumption can be relaxed. However, SMC methods impose a much larger computation burden that may make them prohibitive for imaging large-scale neural systems.

The derivation of the analytic a-priori (prediction through the model) state and parameter estimates provided in this paper gives an exact solution for the expected value for a Gaussian transformed by a sigmoid, regardless of the shape of the resultant distribution. This improves on the the unscented or extended Kalman filters, which have previously been used in a similar context (Voss et al., 2004; Schiff and Sauer, 2008; Liu and Gao, 2013). The Gaussian approximation of the uncertainty in the state and parameter estimates that are predicted by the model is maintained in our framework using the unscented transform.

The implementation of the unscented transform with large covariance matrices is a well established limitation of the filter (Wan and Van Der Merwe, 2000; Simon, 2006; Särkkä, 2013). While scaling up the size of the model did not significantly increase the estimation bias in this case, it does exponentially increase the computation time to the point where it becomes impractical for real-time applications. For increasing numbers of variables to be estimated, the covariance matrix eventually becomes so large that the use of the unscented transform becomes computationally infeasible. The extended Kalman filter is one possible alternative for approximating the covariance, but estimation accuracy is compromised (for the sigmoid nonlinearity). A possible direction of future research is improved methods of covariance estimation.

A probabilistic (Bayesian) approach is also used in the dynamic causal modeling (DCM) framework, which utilizes an expectation maximization algorithm. However, in the DCM framework, individual distributions of states and parameters are not estimated, where uncertainty is placed over the full model including the measurement function. DCM fits a range of candidate models with various inter-region connectivity structures, and then selects the most appropriate candidate using an information theoretic criterion (Daunizeau et al., 2009). DCM has been applied across a range of data from fMRI (David et al., 2008), ECoG time series (David, 2007) and EEG spectral response (Moran et al., 2008), as well as different phenomena such as seizure prediction (Aarabi and He, 2013) and auditory habituation (Wang and Knösche, 2013). A possible advantage of the Kalman (and sequential) filtering approaches over the DCM framework and other similar methods (such as genetic algorithms) is the ability to track slowly changing parameters in real time, which is likely to be particularly important when investigating transitions observed in data, such as epileptic seizures.

The algorithm presented in this paper utilized known constraints of physiological variables. Enforcing constraints on states and parameters greatly improved the convergence properties of the filter. Without any bounds applied to the distributions of parameter estimates, the results typically did not converge to a steady value within the simulation time-frame. There are a number of alternative and more theoretically rigorous approaches for constraining the parameter estimates. However, most constraint methods add a significant computational burden to the filter (Simon, 2006; Kandepu et al., 2008), rendering them impractical for implementation in large-scale systems. The large number of states and parameters to be estimated restricted the constraint method to clipping, which is computationally efficient to implement. Future work in this area should be to investigate effect of constraints on the estimation performance (such as the estimate variance).

The initialization of the filter, in particular the covariance matrix, is a notoriously inexact science (Wan and Nelson, 1997; Wan and Van Der Merwe, 2000; Simon, 2006; Schiff, 2012). In practice, significant tuning is often required to achieve stable and accurate estimation results. For this study, the initial covariance was based on knowledge obtained from forward simulations. A larger initial covariance was used when the number of hidden variables was increased. The initial uncertainty for parameters was increased by broadening the range of the constraints. Furthermore, when parameters to be estimated are dynamic rather than static (as would be the case for most parameters of interest in neural models), an additional constant error term is added to the covariance matrix to prevent an overestimate of confidence in the model (Voss et al., 2004). In this case it was found that additional uncertainty should be very small relative to the magnitude of the parameter. The amplitude of the additive uncertainty is analogous to a learning rate parameter in other algorithms. It can be relatively easily tuned by examining the convergence rate the parameters (i.e., see Figure 9).

The estimation framework presented in this paper can be naturally integrated with other existing imaging technologies and computational methods in the field of neuroscience. All methods of neuroimaging are essentially inversion problems, that rely on a transformation from the measurement space to the source space. An example is the transformation of magnetic radiation to the haemodynamic response in fMRI. Typically, measurements are transformed using a specific inversion technique to determine the state of the neural tissue. The framework presented in this paper applies the same philosophy. However, the transformation from the measurement to the source space is via a generative model. The generative model reflects the current state-of-the-art of our knowledge of the mesoscopic biophysics and anatomy of cortical circuits. By the same token, limitations and uncertainties in our current knowledge can also be quantified and incorporated into the model, making all predictions reflect probability distributions rather than scalar values. The mapping from neural population models to measurements can be readily adapted to describe different modalities, via alternative observation equations, enabling multiple sources of data to be combined to form a unifying model. The difficulty of measuring brain activity in a minimally invasive manner makes it imperative to use as much information as possible to predict neural states and inter-connectivities. A framework that combines patient-specific measurements with well accepted principles of brain structure and function, and importantly, knowledge of uncertainty, is an important step toward the lofty goal of reverse engineering the brain.

The estimation framework presented in this model could be used as the first stage of a seizure prediction system, providing the necessary features that are used as inputs to a classifier. It is necessary to represent neural data using representative features in order to reduce the dimensionality of the problem prior to applying a classification algorithm. In the past, efforts have focused on defining features that are correlated with ictal and pre-ictal periods and, as such, can be used in a predictive capacity (Andrzejak et al., 2001; Lehnertz et al., 2003). Recently a patient-specific seizure classifier for ECoG was implemented using parameters identified from a neural mass model (Aarabi and He, 2013). The advantages of using neural states and parameters as features for seizure classification is that they are naturally patient-specific (since they are directly relatable to the neural activity) and may also provide clues as to the underlying cause of seizures, which could inform treatment strategies.

The capability of neural models to be tailored to an individual patient's data is particularly relevant to the investigation and treatment of epilepsy, since it is a highly patient-specific disorder. The mechanisms for seizure onset and propagation vary significantly between patients (Wendling et al., 2005; Mormann et al., 2007; Coombes and Terry, 2012). Ideally, information about neural interconnectivity should be obtained on a case-by-case basis using an individualized model (Blenkinsop et al., 2012; Nevado-Holgado et al., 2012). A reliable model inversion framework will enable more precise targeting of therapies. The information provided by a model-based framework could also predict the response to drug treatments or electrical stimulation in a simulated environment, sparing a patient the negative side effects that may arise from a trial-and-error approach. Models can also be used to provide feedback for deep brain stimulators for robust prevention of seizures (Mormann et al., 2007; Adhikari et al., 2009).

This paper presented a framework rather than a specific method. Within the framework, the level of realism of the model can be increased to include more neural population subtypes and the spatial extent can increased to model larger cortical networks. The end goal is to provide the tools to create patient-specific models that use all of the available patient-specific neuroimaging data. Existing studies have demonstrated that this framework is capable of being extended to describe more complex phenomena through the inclusion of, for example; more populations and regions (Babajani-Feremi and Soltanian-Zadeh, 2010; Wang and Knösche, 2013), self feedback connections (Ursino et al., 2010) and firing rate modulated plasticity/habituation of synapses (Deco et al., 2008; Moran et al., 2013) or spatially dependent dynamics (Freestone et al., 2011; Aram et al., 2013). As the model size and complexity increases, there will be new parameters that need to be estimated as they are not directly measurable by other means. There are a number of potential directions that should be investigated to address the problem of dimensionality, such as model reduction, improved methods of covariance approximation or linearization techniques. Finally, further validation of the proposed estimation framework on patient data is necessary to evaluate the true predictive capability of this method.

## Author contributions

Dean R. Freestone and Philippa J. Karoly contributed to all aspects of the paper, including conception of ideas, derivation of new analytic results, software development, and testing, interpretation of results, and writing and editing of the manuscript. Philippa J. Karoly led the software development and simulation experiments. Dean R. Freestone led the model and estimation derivations. David B. Grayden, Dragan Nešić, Parham Aram, and Mark J. Cook all contributed toward conceiving the ideas and drafting the manuscript. All authors have provided final approval and are accountable for all aspects of the research.

## Funding

This work was funded by the Australian Research Council (Linkage Project LP100200571).

### Conflict of interest statement

The authors declare that the research was conducted in the absence of any commercial or financial relationships that could be construed as a potential conflict of interest.

## 5. Appendix

### 5.1. Discretization

To begin, we start with the exact continuous time system

(A1) $$\dot{\xi }\;={\left[\begin{array}{cc} \dot{x} & \dot{\theta } \end{array}\right]}^{⊤}\;$$

(A2) $$={\left[\begin{array}{cc} f^{e}\left(x,\theta ,u\right) & 0 \end{array}\right]}^{⊤}$$

(A3) =F(ξ,u).               

Discretization is performed using the Euler method, where the integration time step is denoted by δ by

(A4) $$F_{\delta }^{a}\left(\xi ,u\right)≜\xi +\delta F\left(\xi ,u\right).$$

The approximate discrete time system can be written in the compact form

(A5) $$\xi_{t+1}^{a}=F_{\delta }^{a}\left(\xi_{t},u_{t}\right),$$

where *a* denotes approximate and the subscript δ indicates that the model is parametrized by integration step size. Now, if we let the discrete time system that corresponds to an exact solution to the continuous system at the integration steps be *f^e^*_δ_(**x**_*t*_,**u**_*t*_), then under reasonable conditions it can be proven that the solution to the approximate discrete time system is consistent, such that

(A6) $$\left|F_{\delta }^{e}\left(\xi_{t},u_{t}\right)-F_{\delta }^{a}\left(\xi_{t},u_{t}\right)\right|\leq \delta \rho (\delta ),$$

where ρ(·) is a class-*K* function that has a dependance on size of the set of ξ and *u* (see Arcak and Nešić, 2004 for details). In the body of this paper, we will drop the subscript δ for notational convenience. However, we stress that the discrete time model is an approximation of the continuous system and is parameterized by the integration time step.

### 5.2. Definition of matrices A, B, C, and D

The continuous time system can be written as

(A7) $$\dot{\xi }=A\xi +B\xi ◦g(C\xi )+D(u)\xi$$

where the matrices **A**, **B**, **C**, and **D**(**u**) ∈ ℝ^*n*_ξ_ × *n*_ξ_^ and *n*_ξ_ = 3(*N* + *K*). For a fixed integration time step, δ, the discrete time model can be written in the form

(A8) $$\xi_{t+1}=A_{\delta }\xi_{t}+B_{\delta }\xi_{t}◦g(C\xi_{t})+D_{\delta }(u)\xi_{t}$$

where **A**_δ_, **B**_δ_, and **D**_δ_(**u**) have the same dimension as their continuous time counterparts. (Note ◦ is the element-wise vector product)

In this appendix, we define all the matrices in Equations A7 and A8 and show the relationship between the models. The model contains (*N* + *K*) synaptic connections (*N* local connections and *K* inter-regional connections). Therefore, the number of parameters (connectivity coefficients) is defined as *n*_θ_ = (*N* + *K*) and the number of states (PSPs and their derivatives) is defined as *n_x_* = 2(*N* + *K*).

The matrix **A** has a block diagonal structure that is comprised of two sub-matrices,

(A9) $$A=\left[\begin{array}{cc} \Psi & 0\\ 0 & I_{n_{\theta },n_{\theta }} \end{array}\right],$$

where **I**_*n*_θ_, *n*_θ__ ∈ ℝ^*n*_θ_ × *n*_θ_^ is the identity matrix and Ψ ∈ ℝ^*n*_*x*_ × *n*_*x*_^ is also composed of the sub-matrices;

(A10) $$\Psi \;=\text{ diag}(\Psi_{j})\;$$

(A11) $$\Psi_{j}\;=\left[\begin{array}{cc} 0 & 1\\ -\frac{1}{\tau_{j}^{2}} & -\frac{2}{\tau_{j}} \end{array}\right],$$

where *j* = 1, …, *N* + *K* indexes connections.

The discrete time version **A**_δ_ is related to **A** by

(A12) $$A_{\delta }=\left[\begin{array}{cc} I+\delta \Psi & 0\\ 0 & I \end{array}\right].$$

The matrix **B** has the form

(A13) $$B=\left[\begin{array}{cc} 0_{n_{x},n_{x}} & \Theta \\ 0_{n_{\theta },n_{x}} & 0_{n_{\theta },n_{\theta }} \end{array}\right],$$

where **0**_*n*_θ_, *n*_ ∈ ℝ^*n*_θ_ × *n*^ are zero matrices (for *n* = *n*_*x*_, *n*_θ_). **Θ** ∈ ℝ^*n*_*x*_ × *n*_θ_^ maps the connectivity gains to the relevant sigmoidal activation function and is of the form

(A14) $$\Theta =\left[\begin{array}{ccc} 0 & \ldots & 0\\ \frac{b_{1}}{\tau_{1}} & & 0\\ \vdots & \ddots & \vdots \\ 0 & & 0\\ 0 & \;\ldots & \frac{b_{N+K}}{\tau_{N+K}} \end{array}\right],$$

where *b_j_* = 1 if the relevant connectivity gain is associated with an internal connection, otherwise *b_j_* = 0 (where *u_j_* ≠ 0) and the input is from an external population and is captured in the matrix **D**_δ_(**u**), which is described below. The discrete time version is simply

(A15) $$B_{\delta }=\delta B.$$

The adjacency matrix **C** is the same for both the continuous and discrete version of the model. It has a block diagonal structure where

(A16) $$C=\text{ diag}(\Gamma ,0_{n_{\theta },n_{\theta }})$$

and **Γ** ∈ ℝ^*n*_*x*_ × *n*_*x*_^ sums the relevant post-synaptic potentials to form the mean membrane potentials then maps them to the activation function and is of the form

(A17) $$\Gamma =\left[\begin{array}{ccccc} 0 & 0 & \ldots & 0 & 0\\ \gamma_{2,1} & 0 & & \gamma_{1,n_{x}-1} & 0\\ \vdots & & \ddots & & \vdots \\ 0 & 0 & & 0 & 0\\ \gamma_{n_{x},1} & 0 & \ldots & \gamma_{n_{x},n_{x}-1} & 0 \end{array}\right].$$

The rows of **Γ**, which we will denote by γ_*j*_, index the PSPs that contribute to the mean membrane potential of the presynaptic populations.

The input matrix **D**(**u**) has the structure

(A18) $$D(u)=\left[\begin{array}{cc} 0_{n_{x},n_{x}} & U\\ 0_{n_{\theta },n_{x}} & 0_{n_{\theta },n_{\theta }} \end{array}\right],$$

where the matrix **U** ∈ ℝ^*n*_*x*_, *n*_θ_^ is given by

(A19) $$U=\left[\begin{array}{ccc} 0 & \ldots & 0\\ \frac{u_{1}}{\tau_{1}} & & 0\\ \vdots & \ddots & \vdots \\ 0 & & 0\\ 0 & \ldots & \frac{u_{N+K}}{\tau_{N+K}} \end{array}\right].$$

The inputs *u_m_* are zero for the majority of the elements, where there is only one external input per region in the current formulation. Each active input is a constant value. The discrete time version is

(A20) $$D_{\delta }(u)=\delta D(u).$$

### 5.3. Expectation of a Gaussian membrane potential transformed by a sigmoid

The prediction step in Kalman filter for the neural population model can be solved analytically given the solution of the expected value of the Gaussian membrane potential that is transformed by the nonlinear sigmoidal activation function. The solution for this problem is provided in this appendix. In order to provide the most concise derivation as possible, we will let mean firing threshold parameter *v*_0_ = 0 and firing threshold variance ς = 1. The solution is provided for an arbitrary *v*_0_ and ς, which can be found via the same sequence of steps in the derivation.

Let our Gaussian random variable, *v*, be described by the probability density function

(A21) $$p(v)=\frac{1}{\sigma \sqrt{2\pi }}\exp\left(-\frac{{\left(v-\mu \right)}^{2}}{2\sigma^{2}}\right).$$

The expected value of the Gaussian random variable transformed by the sigmoid is defined by

(A22) $$𝔼\left[g(v)\right]=\;\int_{-\infty }^{\infty }g(v)p(v)\text{d}v$$

(A23) $$\;=\frac{1}{\sqrt{2\pi }}\int_{-\infty }^{\infty }\int_{-\infty }^{v}\exp\left(-\frac{z^{2}}{2}\right)p(v)\text{d}z\text{ d}v.$$

To proceed, we can make the substitution *z* = *w* − *v* to get *v* out of the integral terminal giving

(A24) $$𝔼\left[g(v)\right]\;=\frac{1}{\sqrt{2\pi }}\int_{-\infty }^{\infty }\int_{-\infty }^{0}\exp\left(-\frac{{\left(w-v\right)}^{2}}{2}\right)p(v)\text{d}w\text{d}v.$$

Next we substitute in the equation for the probability density function of the membrane potential and switch the order of integration, which can be changed without altering the limits of integration giving

(A25) $$𝔼\left[g(v)\right]\;=\frac{1}{2\pi \sigma }\int_{-\infty }^{0}\int_{-\infty }^{\infty }\;$$

(A26) $$\;\exp\left(-\frac{{\left(w-v\right)}^{2}}{2}-\frac{{\left(v-\mu \right)}^{2}}{2\sigma^{2}}\right)\text{d}v\text{d}w$$

Now we need to integrate out *v*, so we collect all the *v*-related terms

(A27) $$\begin{array}{l} 𝔼\left[g(v)\right]=\frac{1}{2\pi \sigma }\int_{-\infty }^{0}\exp\left(-\frac{1}{2\sigma^{2}}\left(\sigma^{2}w^{2}+\mu^{2}\right)\right)\\ \;\times \int_{-\infty }^{\infty }\exp\left(-\frac{\sigma^{2}+1}{2\sigma^{2}}v^{2}+\frac{\sigma^{2}w+\mu }{\sigma^{2}}v\right)\text{d}v\text{d}w. \end{array}$$

Integrating out *v* in the second term we get

(A28) $$\begin{array}{l} \int_{-\infty }^{\infty }\exp\left(-\frac{\sigma^{2}+1}{2\sigma^{2}}v^{2}+\frac{\sigma^{2}w+\mu }{\sigma^{2}}v\right)\text{d}v\\ \;=\frac{\sqrt{2\pi }\sigma }{\sqrt{\sigma^{2}+1}}\exp\left(\frac{{\left(\sigma^{2}w+\mu \right)}^{2}}{2\sigma^{2}(\sigma^{2}+1)}\right). \end{array}$$

The solution in Equation A28 is then recombined with Equation A27. After rearranging and simplifying, the expected value becomes

(A29) $$𝔼\left[g(v)\right]=\frac{1}{2\pi }\frac{\sqrt{2\pi }}{\sqrt{\sigma^{2}+1}}\int_{-\infty }^{0}\exp\left(-\frac{{\left(w-\mu \right)}^{2}}{2\left(\sigma^{2}+1\right)}\right)\text{d}w.$$

To solve this last integral, we perform a change of variables

(A30) $$z\;=\frac{w-\mu }{\sqrt{\sigma^{2}+1}},\frac{\text{d}z}{\text{d}w}=\frac{1}{\sqrt{\sigma^{2}+1}}$$

(A31) $$\text{d}w\;=\sqrt{\sigma^{2}+1}\text{d}z,\;$$

giving the final result,

(A32) $$\begin{array}{l} 𝔼\left[g(v)\right]=\frac{1}{\sqrt{2\pi }}\int_{-\infty }^{\frac{\mu }{\sqrt{\sigma^{2}+1}}}\exp\left(-\frac{z^{2}}{2}\right)\text{d}z\\ \;=\frac{1}{2}\left(\text{erf}\left(\frac{\mu }{\sqrt{2(\sigma^{2}+1)}}\right)+1\right). \end{array}$$

The more general solution for an arbitrary mean firing threshold, *v*_0_, and firing threshold variance, ς, is

(A33) $$𝔼\left[g(v)\right]=\frac{1}{2}\left(\text{erf}\left(\frac{\mu -v_{0}}{\sqrt{2\left(\varsigma^{2}+\sigma^{2}\right)}}\right)+1\right).$$

### 5.4. Unscented transform

The sigma vectors are defined as

where κ is a constant that can be tuned which determines the spread of the sigma vectors around the mean and β is a parameter that can be used to incorporate information about the distribution of the states (2 is optimal for Gaussians) (Wan and Van Der Merwe, 2001). The vector ${\left(\sqrt{\left(n_{x}+\kappa \right){\hat{P}}_{t-1}^{+}}\right)}_{i}$ is the *i*^th^ column of the matrix square root (e.g., the lower triangular matrix that can be computed using the Cholesky decomposition), where *i* = 1, …, *n_x_*.

The weights, *W_i_*, for the unscented transform are calculated as

(A37) $$W_{0}\;=\frac{\kappa }{n_{x}+\kappa }+\beta \;$$

(A38) $$W_{i}\;=\frac{1}{2\left(n_{x}+\kappa \right)}\;i=1,\ldots ,2n_{x}.$$

For the initialization of the Kalman filter in this paper, algorithm values were

(A39) β =2          

(A40) $$\kappa \;=3-2n_{x},$$

where *N* is the number of synapses.

### 5.5. Algorithm initialization

To initialize the filter, $\hat{\xi }$^+^_0_ and off-diagonal elements of $\hat{P}$^+^_0_ were set to zero. The diagonal elements of $\hat{P}$^+^_0_ corresponding to fast states (PSPs and their derivatives) were set to the variances of the states obtained from forward simulations. The initial variance estimate for the slow states (connectivity parameters) were set by recognizing that the variance of each PSP in the state vector is proportional to the amplitude of the connectivity parameter that is associated with that particular connection. Therefore, the initial estimation variance for each connectivity parameter was set to be proportional (by a scaling parameter) to the variance of the associated PSP obtained from forward simulation. Scaling parameters were chosen for each connection subtype to reflect the different orders of magnitude of the connectivity strengths (shown in Table A1). The weighting for the slow state $\hat{P}$^+^_0_ values was determined by normalizing across all the regions for connection specific PSPs; i.e., let

(A41) $$\begin{array}{l} \beta ≜\left[\begin{array}{ccccccc} \text{var}(v_{up}^{1}) & \text{var}(v_{ep}^{1}) & \text{var}(v_{pi}^{1}) & \text{var}(v_{ip}^{1}) & \text{var}(v_{pe}^{1}) & \text{var}(v_{jk}^{1}) & \text{var}(v_{kj}^{1})\\ \vdots & & & & & & \vdots \\ \text{var}(v_{up}^{J}) & & & \cdots & & & \text{var}(v_{kj}^{J}) \end{array}\right]\\ \;=\left[\begin{array}{c} \Sigma_{v}^{1}\\ \vdots \\ \Sigma_{v}^{J} \end{array}\right] \end{array}$$

for *J* cortical regions. The normalized matrix is given by

(A42) $$M=\text{diag}\left(∥\Sigma_{v}^{1}{∥}_{\infty }^{-1},\ldots ,∥\Sigma_{v}^{J}{∥}_{\infty }^{-1}\right)\beta ,$$

where we are normalizing using the *L*_∞_ norm of each of the rows of β, which are denoted by **Σ***^j^_v_*. The resultant matrix **M** is scaled to form the initial values of the variances for the connectivity estimates. The scaling values to set the values of $\hat{P}$^+^_0_ are shown in Table A1.

**Table A1:** **Initial values for the elements of $\hat{P}$^+^_0_ that correspond to connectivity gain estimates**.

**Parameter**	**Initial variance**
α_*up*_	0.1 **M**_*j*,1_
α_*ep*_	10 **M**_*j*,2_
α_*pi*_	1 **M**_*j*,3_
α_*ip*_	60 **M**_*j*,4_
α_*pe*_	10 **M**_*j*,5_
α_*jk*_	5 **M**_*j*,6_
α_*kj*_	5 **M**_*j*,7_

*The matrix **M** is derived from the PSP variances from a forward simulation and *j* = 1, …, *J* indexes the cortical region*.

To initialize the filter values for the model and measurement variance in the Kalman filter equations (denoted **Σ** and **R**, respectively) knowledge of the forward simulation was used. The measurement variance was set to

(A43) $$R=\sigma_{y}^{2}I_{n_{y},n_{y}},$$

where σ_*y*_ is the standard deviation of the additive measurement noise used in the forward simulation for the ECoG signal, which was 1 mV. **I**_*n*_*y*_, *n*_*y*__ is the identity matrix and *n_y_* is the number of measurements (i.e., the number of regions in this case).

The model uncertainty was set to

(A44) $$\Sigma =\{\begin{array}{ll} 10^{-16}I_{n_{\xi },n_{\xi }}+Q & \text{for static parameters}\\ 10^{-16}I_{n_{\xi },n_{\xi }}+Q+Q^{\theta } & \text{for parameter tracking} \end{array},$$

where the first term on the left hand side is for numerical stability, **Q** is the known covariance matrix of process noise, **w**_*t*_, that was used in the forward simulations, and the **Q**^θ^ term represents a constant additive covariance for parameter tracking purposes,

(A45) $$Q^{\theta }=\text{diag}(0_{n_{x},n_{x}},\Sigma^{\theta }).$$

When the filter is used to track parameter dynamics, **Σ**^θ^ is used to capture the unexpected changes (this is not necessary for the state as their dynamics are modeled, whereas parameters are assumed to be static by the filter). **Σ**^θ^ was a diagonal matrix, where for *j* = 1 … *n*_θ_,

The  notation shows that the uncertainty is proportional to the order of the connectivity gain (α_*j*_). The coefficients can be tuned to adjust the rate of estimation convergence. The smaller value for α_*up*_ was the result of tuning based on the estimation results.
